# Managing ‘shades of grey’: a focus group study exploring community-dwellers’ views on advance care planning in older people

**DOI:** 10.1186/s12904-016-0175-7

**Published:** 2017-01-13

**Authors:** Natasha Michael, Clare O’Callaghan, Emma Sayers

**Affiliations:** 1 Palliative Care Research Department, Cabrini Institute, 154 Wattletree Road, Malvern, VIC 3144 Australia; 2 School of Medicine, University of Notre Dame Australia, 160 Oxford Street, Sydney, NSW 2010 Australia; 3 Faculty of Medicine, Nursing and Health Sciences, Monash University, Melbourne, VIC 3800 Australia; 4 Department of Medicine, St Vincent’s Hospital, The University of Melbourne, 41 Victoria Street, Fitzroy, VIC 3065 Australia; 5 Optimal Care Pathway, Royal Children’s Hospital, 50 Flemington Rd, Parkville, VIC 3052 Australia

**Keywords:** Advance care planning, Aged, Qualitative research, Community health services, Caregivers

## Abstract

**Background:**

Community-dwelling consumers of healthcare are increasing, many aging with life-limiting conditions and deteriorating cognition. However, few have had advance care planning discussions or completed documentation to ensure future care preferences are acted upon. This study examines the awareness, attitudes, and experiences of advance care planning amongst older people and unrelated offspring/caregivers of older people residing in the community.

**Methods:**

Qualitative descriptive research, which included focus groups with older people (55+ years) and older people’s offspring/caregivers living in an Australian city and surrounding rural region. Data was analysed using an inductive and comparative approach. Sampling was both convenience and purposive. Participants responded to web-based, newsletter or email invitations from an agency, which aims to support healthcare consumers, a dementia support group, or community health centres in areas with high proportions of culturally and linguistically diverse community-dwellers.

**Results:**

Eight focus groups were attended by a homogenous sample of 15 older people and 27 offspring/caregivers, with 43% born overseas. The overarching theme, ‘shades of grey’: struggles in transition, reflects challenges faced by older people and their offspring/caregivers as older people often erratically transition from independence and capacity to dependence and/or incapacity. Offspring/caregivers regularly struggled with older people’s fluctuating autonomy and dependency as older people endeavoured to remain at home, and with conceptualising “best times” to actualise advance care planning with substitute decision maker involvement. Advance care planning was supported and welcomed, x advance care planning literacy was evident. Difficulties planning for hypothetical health events and socio-cultural attitudes thwarting death-related discussions were emphasised. Occasional offspring/caregivers with previous substitute decision maker experience reported distress related to their decisions.

**Conclusions:**

Advance care planning programs traditionally assume participants are ‘planning ready’ to legally appoint a substitute decision maker (power of attorney) and record end-of-life treatment preferences in short time frames. This contrasts with how community dwelling older people and offspring/caregivers conceive future care decisions over time. Advance care planning programs need to include provision of information, which supports older people’s advance care planning understanding and management, and also supports offspring/caregivers’ development of strategies for broaching advance care planning with older people, and preparing for potential substitute decision maker roles. Development and integration of strategies to support older people’s decision-making when in the ‘grey zone’, with fluctuating cognitive capacities, needs further consideration. Findings support an advance care planning model with conversations occurring at key points across a person’s lifespan.

## Background

Community care is defined as providing the right level of intervention and support to enable people to achieve maximum independence and control over their lives [1]. This is particularly relevant to the increasing number of older people (OP) now living in the community. Many experience chronic and life-limiting illnesses such as dementia, cancer, and progressive neurological conditions, which are associated with, slow decline, and sporadic exacerbations of illness and disability [2]. Initial adverse impacts of these diseases mostly originate in community settings, thus requiring community-dwelling OP, their families, and caregivers to be increasingly involved in healthcare decision-making.

Healthcare strategies incorporating quality initiatives for chronic diseases and end-of-life (EOL) care in the last year of a patient’s life emphasise the need for engagement in advance care planning (ACP) [3]. ACP is a process of reflection that enables people to consider their values and goals and subsequently share them with clinicians and relevant others [4]. ACP should be considered over time and may be communicated or documented to uphold individuals’ wishes for such time that they may lose capacity to make informed decisions [5]. ACP assists clinicians challenged by treatment cessation decisions, particularly in OP where extended life expectancies may be associated with co-morbidities and increasing frailty [6]. Further benefits include improved patient satisfaction, quality of life, survivors’ mood and adjustment, earlier hospice admissions [7, 8], and fewer hospitalizations from nursing homes [9].

Despite increasing numbers of OP living in communities [10], studies have demonstrated that up to 70% facing EOL are incapable of care related decision-making [11], suggesting that early care planning in OP should be prioritized. In a UK study of over 1800 community dwelling people over 65-years-old, only 17% had prepared an ACP document, and many had rarely discussed future care plans with doctors, instead preferring ACP discussions with families [12]. Interviews with 37 English community-dwelling OP (mean 71.4 years) revealed that they also rarely documented healthcare plans, but often had formal financial and funeral plans. Individual inclinations affected openness to planning, with some just ‘liv(ing) for today’. Poor ACP related legal literacy and understanding about accessing assistance available were also common [13].

In Australia, with the promotion of the National Framework for Advance Care Directives, 14% of the general population over the age of 18 have now prepared ACP documents, although marked variation exists across states [14]. Recent research in one metropolitan Australian city found that 27% of nursing home residents (mean 86.2 years) had documented ACPs [15]. The nursing home residents’ plans were often ambiguous, difficult to interpret, and sometimes overridden [15]. More recently, an Australian national government funded initiative, *Decision Assist,* has been promoted to promote ACP by improved linkages between aged and palliative care services via a phone advisory service [16]. Further Australian research found that attitudes towards ACP varied amongst migrant groups with first generation Italian migrants preferring decision-making styles that involved family members collectively (together) and Dutch migrants preferring a more individualistic approach [17].

OP’s carers’, and family members’ views are needed because of their important roles in supporting individuals’ ACP, particularly within community settings [5, 18]. ‘Listening events’ conducted across the UK to understand EOL concerns amongst OP and caregivers found that many welcomed opportunities to discuss ACP, including those participants from ethnic groups whose desire for information often superseded fears related to ‘bad luck’ that may follow such discussions [18]. However, North American findings indicate sub-optimal agreement between OP and proxies about EOL care communications [19]. Improvements in how older community dwellers prepare for EOL are needed, and ACP programs should reflect needs and experiences of those anticipated to use related services [20].

This research evolved from a project aimed at encouraging consumer participation in ACP. As the majority of respondents were at least 55-years-old, this study specifically aimed at gauging ACP awareness, attitudes, and experiences amongst OP, and unrelated offspring/caregivers of OP residing in the community.

## Methods

A qualitative descriptive research (QDR) design with ‘grounded theory overtones’ [21 p. 337] was used. QDR can include analytical strategies informed by grounded theory [21, 22]; it does not directly enable theory generation because it does not use theoretical sampling [21]. Participants were recruited between March and April 2015 via the Health Issues Centre, a not-for-profit, partly government funded agency, which aims to support and inform consumers and the health sector of healthcare improvements. Sampling was purposeful, targeting community dwelling participants, including OP (aged-55-or-over) with/without chronic or serious illness, and unrelated offspring/caregivers of OP with/without chronic or serious illness. Initially, participants responded to general invitations to participate via the Health Issues Centre’s website. To ensure diverse perspectives, participants were additionally sourced through newsletters or emails distributed by a dementia support group and local community health centres in locations with high proportions of culturally and linguistically diverse community-dwellers. Potential participants were invited to discuss experiences of future healthcare planning. The phrase, ‘ACP’, was not used, as previous research indicated local unfamiliarity with the term [5].

Offspring/caregivers were aged over 18 and not related to older adult participants. Participants were required to have good English comprehension and cognition. In defining the cut of age for inclusion, the research team considered the standard Australian definition of OP [23], as well as socially constructed meanings of OP, such as the loss of roles accompanying physical decline, and variation in classification of an OP between countries [24]. As such, 55 was set as the cut-off age for inclusion into the study.

Eight focus groups of between 5–7 participants were conducted by ES and CO, with one group of offspring/caregivers requiring interpreter assistance with a Cantonese interpreter. Five groups were conducted in meeting rooms at the Health Issues Centre and three groups in community health centres. Groups for OP and offspring/caregivers were held separately.

Following consent and demographic detail collection, focus group discussions were conducted using a semi-structured question framework. Questions focused on participants’ understandings and views about ACP; related discussion or documentation experiences; future health concerns for themselves or those being cared for; and experiences related to planning future healthcare. Discussions were recorded and transcribed.

Focus group transcripts were imported into qualitative data management software where they were initially analysed separately as two subgroups: OP and offspring/caregivers of OP. Comparative and cyclic data analysis included inductive line-by-line coding and amalgamation of comparable codes into researcher-created categories. Comparable categories across both subgroups were amalgamated into ‘major categories’. Comparable major categories were then grouped into themes. CO conducted initial analyses of subgroup data. Qualitative inter-rater reliability proceeded, whereby ES and NM read transcripts and these initial analyses. CO, ES, and NM (all experienced qualitative researchers) exchanged analytic views until satisfied with the representations of the subgroup data (categories). After CO developed the major categories and themes, ES, NM, and CO also discussed this analysis and the findings were reworked until all were satisfied with the final statement of findings. Thematic analysis illustration is demonstrated in Table 1.

**Table 1:** Illustration of comments, which informed major categories (italics) and themes (bold)

Older People’s comments	Offspring/caregivers’ comments
**Theme 1. ‘Stages of grey’: challenges struggles in transition**	
*1.a. Approaching challenging transitions* Why can’t we go to rehab before the operation to know what will be happening in rehab? It’s sort of, that’s what I call advanced care. (female, 65–74-years-old) *1.b Making sense of advocacy* I told them (Chinese older people) I’ve been here (community centre) and I listen about this, about this type of planning, but sometimes they’re not ready, because by my mouth Chinese, speaking Chinese, they not believe. They need someone (government official) to come and talk to the community like that and they more believe like that. If I told them they said, ‘Maybe’ … But I try my best. (female, 55–64-years-old)	We made that decision, me and my wife, made and just said no, we aren’t going to tell her (wife’s mother) that she’s on the steroids because if we do, she won’t take them. She needs them. (male, 45–54-years-old) It's hard to know someone who has been strong and independent all their life, how much input they should have and are capable of having, depending on which day it is that you're asking. (female one, 55–64-years-old)
**Theme 2. ACP literacy and communication** 2.a. *ACP knowledge and experience* Doctors haven’t raised it (ACP) with me. … and I’ve been told that I was a month away from being dead if I didn’t get a transplant. (male, 55–64-years-old) *2.b. Sociocultural attitudes towards death.* There was just an expectation that we’d all look after one another. (female, 75 + −years-old)	I just thought it meant either turning the switch on or off basically. I didn't realise that it was all to do around medication and things like that. (female two, 55-64-years old) Yeah, some old people (in Chinese culture) just want to live, even though they can’t move, they can’t even get off the bed – they still can eat something, they still want to have a life. (Focus group with interpreter; unable to determine participant)
**Theme 3. Challenges contemplating mortality** 3.a. *Previous life and health experiences* I keep putting it (ACP) off. And I’m put off even more when I look at and aware of what occurs in elder abuse, …, or children often, grabbing that power of attorney very quickly in order to make sure that the house or the money comes to them. (male, 65–74-years-old) *3.b. Planning for hypothetical circumstances* You couldn’t cover everything because we don’t know what’s going to happen to us, do we, in the hospital; anything could happen, so we can’t cover every illness or outcome. (female, 66–64-years-old)	My first thing would be that somehow they find a way to get his kidney working better. Right? So that there’s no need for anything else. … It’s hard work trying to keep him encouraged and to try and look forward to have things. And my view would be that I’d just like him to go in his sleep one night. (female, 65–74-years-old) (Father said) ‘If something happens to your mother I now want to go in somewhere else and sell the house.’ Well, that was a complete change from what he had told me five years ago. So what … if you lock yourself into too tight an advance care plan (and) things change? (female, 45–54-years-old)

*ACP*: advance care planning

## Results

### Participant Characteristics

Participants numbered 42 in total, comprising 15 OP (3 groups) and 27 offspring/caregivers^1^ (5 groups) and lasted 85–105 min. OP groups’ majority were aged between 55 and 64 with 3 aged 65–74 and 3 aged 75+ years. Offspring/caregivers included six partners, one sibling and 20 offspring/in-laws. Participants were born within (57%) and outside (43%) Australia. Detailed demographics are provided in Table 2.

**Table 2:** Characteristics of participants

		Older People (*n* = 15)	Offspring/caregivers (*n* = 27)
Age			
	18–34		2
	35–44		1
	45–54		11
	55–64	7	8
	65–74	5	4
	75+	3	1
Born			
	Australia	10	14
	United Kingdom	1	3
	China		3
	Libya	1	
	Republic of Ireland	1	
	Malaysia	1	
	Vietnam	1	
	Germany		1
	Canada		1
	Poland		1
	Italy		1
	Singapore		1
	Ethiopia		1
	Did not state		1
Relationship to older adult			
	Daughter		18
	Partner		6
	Son/son-in-law		2
	Sister		1

An overarching theme emerged, ‘*shades of grey’: struggles in transitions*. This reflects the challenges faced by both OP and their offspring/caregivers in contemplating ACP when the OP is in the ‘grey zone’ of ageing; transitioning from independence to dependence and capacity to incapacity. Additionally themes were ACP literacy and communication, and challenges contemplating mortality.

### ‘Shades of grey’: struggles in transitions

#### Approaching challenging transitions

Several OP recollected struggling with losses related to their current health conditions as they aged, alongside trying to prepare for the future. Some contemplated that ACP may provide the opportunity to prepare for a state of declining capacity and functionality, stating:

*“Where can I go, to access services?… that’s advanced care planning… autumn of your life…Living well and hopefully preventing some of the issues that currently ageism I think is.”* (female, OP, 65–74-years-old)

Others queried ‘best times’ for ACP actualisation and substitute decision maker (SDM) involvement, as they struggled to ‘figure out’ when one formally ‘crosses the line’.

*“The difficult thing … It's identifying that point where you've got to say that's enough… life isn't black and white. You can't just go from one day to the next and now today is the day that we're going to enact the plan. It's the stages of grey between where you are.”* (female, daughter, 55–64-years-old)

One daughter’s attempts to prepare a participant through this transition by suggesting a number of options, including an assessment for residential care, triggered the older person’s response, *“I may as well jumped off a bridge, I was so disgusted”.*

**Fig. 1:**
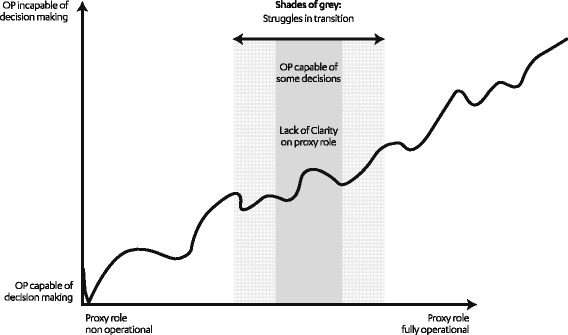
Representation of fluctuating cognition on decision-making and proxy role

Many agreed that the SDM role is complex and vacillates, as OP’s conditions, cognition, and decisional capacity can deteriorate in a fluctuating manner. This is graphically illustrated in Fig. 1.

#### Making sense of advocacy

Many offspring/caregivers were informal advocates, acting on behalf of health and social support systems to assist ageing OP. Offspring/caregivers also regularly said that vulnerable OP they cared for declined community-based supports. Further, offspring/caregivers could struggle to access OP’s financial or medical information when trying to understanding their needs and entitlements. Though dealing with OP through these “shades of grey” posed stressors, some adopted strategies to help them cope as caregivers while transitioning to likely SDM. This included acceptance of OP’s excessive demands, or assessing how to respond to OP’s fluctuating autonomy and dependency, as needed. To prepare for the SDM role, individuals also involved a patient advocate (an independent paid/unpaid professional to assist them in achieving health care outcomes), completed dementia care-related training, sought information from health professionals, and attended OP’s medical appointments when allowed.

Some participants specifically recommended that health professional support for OP and offspring/caregivers was as vital as information provision on ACP as OP became frailer and more dependent in the community setting.

*“You’ve got a maternal healthcare line, you’ve got, I don’t see that there is an ageism care line.”* (female, OP, 55–64-years-old)

### ACP literacy and communication

#### ACP knowledge and experience

All group discussions began with the question, ‘Have you heard the term ACP and what do you think it means?’ The most commonly mentioned terms were *“will:”* and *“power of attorney”*, with limited understanding of ACP from a legal transactional or EOL care planning perspective. Others had some familiarity with financial, but less so with medical power of attorney, and found terminologies confusing. Most required clarification around the different types of attorney and the process of appointment.

*“I haven’t actually heard of it (the term, ACP) … My daughter is my power of attorney but I haven’t done the sickness one.”* (male, OP, 65–74-years-old)

Participants’ understanding arose from ad-hoc social, family, and workplace interactions, seniors’ events, websites, or parents’ healthcare experiences. An OP speculated that 30–50% in his retirement village did not have family, but added, “*We’ve made it our family*”, and that they *“frequently”* discuss ACP related topics. Although only a small number had legally appointed a SDM (called, “medical enduring power of attorney” in Victoria, Australia), many OP had sporadically conversed with others about future medical treatments, residential care plans, and wills. Some OP expressed frustrations that partners would not participate in such discussions. While some offspring/caregivers had shared-understandings and formalised statements to direct SDM roles, some remained uncertain about role execution, sometimes with distressing consequences.

*“With my mother-in-law … dementia wasn't on my radar.... We had to make some pretty serious decisions very quickly because she was interstate … documents between states are different …. They found three aged care facilities. And we had the 10 min to say yes or no.... It was just a nightmare.”* (male, partner, 55–64-years-old) *Sociocultural attitudes towards death*

Understanding of ACP and related communication was also affected by socio-attitudinal reactions towards death. Longstanding cultural death ‘taboos’ within some families presented challenges, though some suggested that this was dissipating, especially amongst OP from Asian backgrounds, with funeral discussions now *“quite a popular topic”*.

Participants’ responses highlighted the spectrum of family relationships and dynamics that people bring to EOL planning. Agreeing to undertake a SDM role could be motivated by a sense of duty, filial attachments, or even guilt. Clashing values and mistrust sometimes featured as a concern that may complicate execution of roles.

*“So we sorted out that he (husband) was not going to approach it the way my daughter and I would, and she would be more likely to obey my wishes than he would.”* (female, OP, 75-plus-years-old)

After acting as a SDM, participants could feel satisfied or troubled, notably when insufficiently medically informed.

*“Well, what do you recommend? You're the doctor. I'm not.’ And he said, ‘Well, I think he should go back on the Aricept.’ I said, ‘Okay’ … And he spent two-and-a-half years in the locked dementia ward … that was disgusting. … I've had a lot of psychosomatic problems with depression. But if I had that piece of paper (ACP document) it would have been easier.”* (male, partner, 65–74-years-old)

While some OP were hesitant to ‘burden’ offspring with ACP involvement, another argued that ACP alleviated decision burden, *“that could affect them for life”*. Attitudes towards ACP documentation also varied. While some believed that documents could *“prove”* they were fulfilling carer’s wishes, others regarded ACP documentation as too formal or *“complicated”.*

### Challenges Contemplating Mortality

#### Previous life and health experiences

Stories and descriptions offered highlighted the significance of prior life experience when contemplating mortality. ACP triggered many reflections, including the influences of relationships, values, and culture. Negative experiences related to ageing, care, and deaths of significant others were particularly influential. In a number of situations, the death of a relative allowed for insight into the practical value of planning.

*“So we have got a plan, it’s not formalised, but we’ve spoken, I suppose in our situation, the reason why we spoke with her was because we’d gone through my father’s death.”* (female, daughter, 55–64-years-old)

In other instances, negative perceptions of life in care sometimes triggered a desire to avoid life-extending medical interventions, and even intensified views regarding a life not worth living.

*“Your life’s finished when you’ve gone there…. I reckon 99% of the people in nursing homes would love to die.”* (female, OP, 75-plus-years-old)

Where optimism and hope had been key tools for survival, planning for incapacity may be resisted. For example, one carer whose husband had long-standing kidney disease, was surprised and distressed by the introduction of an ACP; which she considered unnecessary because the focus always remained on keeping him alive.

#### Planning for hypothetical circumstances

There was much reflection on the conundrum of the ‘in advance’ decision and its relation to the difficulties of imagining incapacity and its consequences. On the one hand, death was accepted as inevitable, with participants envisaging states worse than death and acknowledging that planning *“makes sense”’*. Conversely, consideration of hypothetical options frequently prompted the response that it depended on the circumstances. A wife who had experienced a change in her husband’s desire for life extension was particularly uneasy.

*“(Husband) had a bit of a health crisis about 12 months ago. … when he came out of this acute crisis, he said to me he was afraid people that people would turn off the switch. So there was a complete change of his limited understanding … (now) I don't know whether I'm actually fulfilling his wishes.”* (female, partner, 55–64years-old)

The lack of context and temporality of EOL trajectories was a source of apprehension. Some offspring/caregivers were concerned about being locked into decisions, especially if they were recorded in writing without real time knowledge of particular circumstances.

*“It’s really difficult to get that clarity around it. I feel as though even if it was written out in a document … life isn’t that black and white.”* (female, daughter, 55–64-years-old)

Some offspring/caregivers wrestled with the fear that their loved one would suffer for decisions they could make in ignorance. Their stories revealed a sense of responsibility for making the right decision and doing *“the best thing’*. OP could also identify a need or expectation that someone appropriately knowledgeable would take the lead and guide them through key considerations.

*“Who helps you do it? What do you write down? Is there anyone to guide you do that? Can you get advice from somebody about that?”* (male, OP, 65–74-years-old)

## Discussion

ACP programs traditionally commence with the assumption that participants are ‘planning ready’, with knowledge, skills, and cognition available to proceed with the two central elements: appointment of a SDM, and recording of preferences regarding specific treatments and interventions. This study, however, supports claims that though ACP is well received in the community amongst OP and their offspring/caregivers; personal, relational, and socio-cultural aspects should mitigate consideration of ACP programs focussed on document completion in short timeframes [17, 25]. This study’s findings reinforce that many people consider ACP components over time [17, 26]. Barriers may include predicting future health decisions [17], low health literacy around ACP and associated legal parameters within community settings [7, 27], and difficulties talking about death [27]. Furthermore, when hope and optimism have been used as longstanding coping mechanisms, offspring/caregivers can be challenged in undertaking the SDM role of understanding OP’s future care wishes and planning for their possible incapacity.

This study also highlights that ACP programs for community-dwelling OP and offspring/caregivers requires accommodation of OP’s variable and often fluctuating cognitive capacities and competency spectrums. Specific concerns in ACP may arise when OP enter the capacity ‘grey zone’ (Fig. 1), with fluctuating yet overall declining cognition and executive functioning (reasoning and understanding decisional consequences), and increasing frailty. Educational interventions such as written memory aids [28] may be needed to help these vulnerable OP to better understand the value of ACP and future planning.

Given that variants of OP’s incompetence and incapacity may arise in different situations, flexibility of legal considerations and responses is required [29]. Underpinning this recommendation is the need to respect the dignity of the OP, whose competence is fluctuating, with protection of remaining capacity as a starting point. Offspring/caregivers’ concerns about fluctuating cognition and a slow deterioration suggest that a relational and contextual approach to capacity assessment is needed when OP are in the capacity/incapacity ‘grey zone’. This is comparable to a process consent methodology in dementia research [30]. This acknowledges that capacity is situational, potentially present when a legal threshold is crossed, and often strengthened within caring relationships. It involves critically reflecting on whether the person is consenting, has informed appreciation of consent, and whether lack of objection is genuine.

As the numbers of OP living in the community increase, the legal issue concerning the incompetent older person becomes more relevant. Though the formal nomination of a SDM or representation by next of kin (commonly a partner or family member) may seemingly be a simple process, it has the potential to mask the complex reality of medical decision-making on behalf of incompetent OP. Proxy decisions may be made based on personal motivations, highly charged emotions, and with lack of supervision [31]. In addition, stories presented by offspring/caregivers in this study reflected relative ignorance and anxiety about managing the SDM role delineation when the OP’s competency was uncertain, that is, when and how to become involved in which decision-making areas. Development of interventions, which support SDMs’ preparation for future decision-making, is an important area for future inquiry.

Overall, this study’s findings reinforce the importance of ACP but suggest that ACP programs should accord with the ‘life-cycle model’. Such a model proposes that aspects of advance care planning should occur throughout the continuum of the human life cycle. Discussions about life values and goals should commence at key life maturation points such as turning 18, being married or starting a family having children, and throughout primary health care in the community, and with the diagnosis of a serious illness and its progression [7]. Such a process normalizes ACP and reduces emotional burden that may otherwise arise in those who have not adequately prepared for making end-of-life care decisions. [7]. An ACP life-cycle model should encompass broad information and support, focussing on: (a) planners’ understanding about why ACP is helpful and how they can manage the process; (b) how offspring/caregivers can broach ACP with OP and cope when OP persistently decline discussions; (c) SDM understanding of strategies for managing OP’s process consent when cognition is fluctuating, and (d) assisting with distress sometimes experienced following substitute decision making. ACP programs also need to accommodate diverse individual and collectivist decision-making styles used by planners to determine meaningful issues, goals, and preferences [25].

### Limitations and recommendations

Findings offer insight into how ACP may be considered by community-dwelling OP and offspring/caregivers. However, they only reflect views of those able to consider EOL care, attend focus groups, and have mostly Anglo-Saxon or Asian origins. Further research avenues may consider concerns about ACP ‘non-discussants’ and preparedness for SDM, including how best to approach decision-making with OP within the indeterminate capacity/incapacity ‘grey-zone’.

## Conclusion

Debates about best ACP approaches seldom include evidence about how people consider the meaning of ACP, or their preferences about approaching it [20]. This is especially evident for proxies potentially actualising individuals’ ACPs. Community-dwellers depict ACP as a relational process for OP, reflecting a matrix of individual, family, social-cultural, and systemic factors affecting motivations to discuss and complete related documents. Typical ACP programs which ‘step’ consumers through documents usually assume that consumers are ‘ready’ to plan all components, when this is in fact seldom the case. Plausibly, many current ACP programs undermine the true issues faced by OP and their offspring/caregivers as they face the uncertainty of the in-between space of living and dying [20].

## Abbreviations

ACP: Advance care planning
EOL: End-of-life
OP: Older people
QDR: Qualitative descriptive research
SDM: Substitute decision maker

## Footnotes

1 Three younger people with chronic/serious illnesses also attended one focus group but their views are not included. Included were views from one offspring/caregiver caring for two parents, now deceased, and one OP caring for an adult offspring with Downs Syndrome.
